# ETV2 expression increases the efficiency of primitive endothelial cell derivation from human embryonic stem cells

**DOI:** 10.1186/s13619-014-0014-3

**Published:** 2015-01-07

**Authors:** Anne G Lindgren, Matthew B Veldman, Shuo Lin

**Affiliations:** Department of Molecular Cellular and Developmental Biology, University of California, 615 Charles E. Young Drive South, Los Angeles, CA 90095 USA

**Keywords:** Human embryonic stem cells, Endothelium, ETV2, Differentiation

## Abstract

**Background:**

Endothelial cells line the luminal surface of blood vessels and form a barrier between the blood and other tissues of the body. Ets variant 2 (*ETV2*) is transiently expressed in both zebrafish and mice and is necessary and sufficient for vascular endothelial cell specification. Overexpression of this gene in early zebrafish and mouse embryos results in ectopic appearance of endothelial cells. Ectopic expression of *ETV2* in later development results in only a subset of cells responding to the signal.

**Findings:**

We have examined the expression pattern of *ETV2* in differentiating human embryonic stem cells (ESCs) to determine when the peak of *ETV2* expression occurs. We show that overexpression of *ETV2* in differentiating human ESC is able to increase the number of endothelial cells generated when administered during or after the endogenous peak of gene expression.

**Conclusions:**

Addition of exogenous *ETV2* to human ESCs significantly increased the number of cells expressing angioblast genes without arterial or venous specification. This may be a viable solution to generate *in vitro* endothelial cells for use in research and in the clinic.

## Findings

Embryonic stem cells (ESCs) are pluripotent and thus have the ability to differentiate into all cell types of the body. Previous groups have derived endothelial cells, the cells that line blood vessels, from mouse ESCs (mESCs) and human ESC (hESCs) and shown that they are functional in *in vivo* assays [1-7]. In many differentiation protocols, only a small percentage of cells differentiate into endothelial cells; however, large quantities of cells are required for potential downstream bioengineering applications [1,2].

Expression of Etv2 is required for the proper formation of the vasculature in fish and mammals [8-10]. Overexpression of *Etv2* in differentiating mESCs has been shown to be effective at increasing the number of endothelial cells [9,11]. Inducible expression of *Etv2* over a short period of time concurrent with endogenous expression is sufficient to increase the population of endothelial cells from 8% to 70% [9]. Recent work infecting hESCs with *ETV2* expressing virus showed that roughly 40% of the infected cells could become endothelial-like under modified culture conditions that also support hESC self-renewal [12].

**Figure 1:**
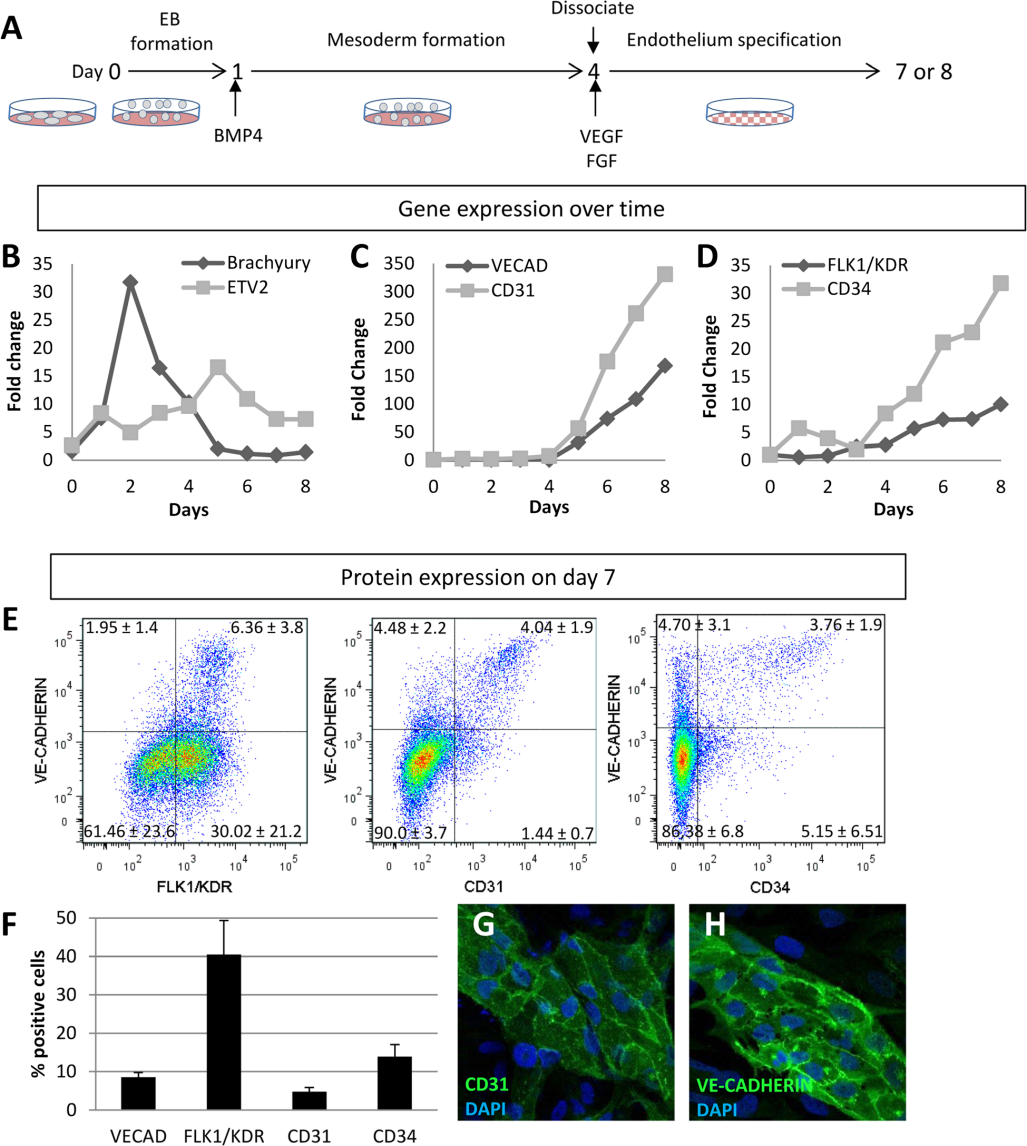
**Differentiation of hESC to endothelial cells. (A)** Diagram of the differentiation protocol. **(B–D)** Semi-quantitative real-time PCR analysis of gene expression in cells from days 0 to 8 of differentiation. Genes examined: **(B)** *BRACHYURY* and *ETV2*. **(C)** *VE-CADHERIN* and *CD-31*. **(D)** *KDR* and *CD34*. **(E)** Flow cytometry analysis of day 7 differentiation of hESC. Percentages shown represent averages from five experiments with standard deviations. **(F)** Quantitative analysis of data in panel **(E)**. Immunofluorescence of day 7 of differentiation for CD31 **(G)** and VE-CADHERIN **(H)**.

We wanted to determine if addition of exogenous *ETV2* during differentiation could induce endothelial cells from hESCs more effectively than addition before differentiation. First, we determined the timing of the expression of endogenous *ETV2* in a hESC differentiation model. hESCs were differentiated into endothelial cells using a method that utilized both embryoid body (EB) and adherent stages and were similar to those reported previously (Figure 1A) [1,3]. The cells were collagenase IV digested into clusters and allowed to form EBs overnight in mTeSR1 media in low adherence plates for 24 h. The EBs were collected by gravity and the medium was replaced with mTeSR1 supplemented with 10 ng/ml BMP4. Four days later, the EBs were digested to single cells with Accutase and plated on Matrigel-coated plates in DMEM/F12 media supplemented with 15% KSR, 25 ng/ml VEGF and 20 ng/ml bFGF2. To determine the timing of gene expression, we collected RNA samples from days 0 to 8 of hESC differentiation. Semi-quantitative real-time PCR performed on cDNA generated from the extracted RNA showed that *BRACHYURY* expression, a marker of mesoderm specification, peaked on day 2 while *ETV2* expression peaked on day 5 of differentiation (Figure 1B). This is comparable to the timing of the expression of *Brachyury* and *Etv2* in the mesoderm of mice, where the *Brachyury* expression precedes a wave of *Etv2* expression by 2 days [9,13,14]. The endothelial markers *VE-CADHERIN*, *CD31*, *KDR*, and *CD34* showed an increase on day 5 that continued for the next 3 days (Figure 1C,D).

To determine the percentage of endothelial-like cells, we analyzed the surface expression of VE-CADHERIN/CDH5, CD31, FLK1/KDR, and CD34 on day 7 of differentiation by flow cytometry. The greatest number of cells expressed KDR (40.4%) (Figure 1E,F). This agrees with previous reports in the mouse and human systems where KDR marked endothelial cells as well as a large population of mesodermal precursors and undifferentiated hESCs [15,16]. VE-CADHERIN (8.5%), CD31 (4.8%), and CD34 (13.8%) were expressed on similar-sized populations of cells and the majority of these cells showed overlap with the three markers (Figure 1E,F). To determine if the cells differentiated in clusters or from scattered single cells, we stained the cells *in situ* on day 7 of differentiation. Clusters of CD31 and VE-CADHERIN cells were seen (Figure 1G,H).

**Figure 2:**
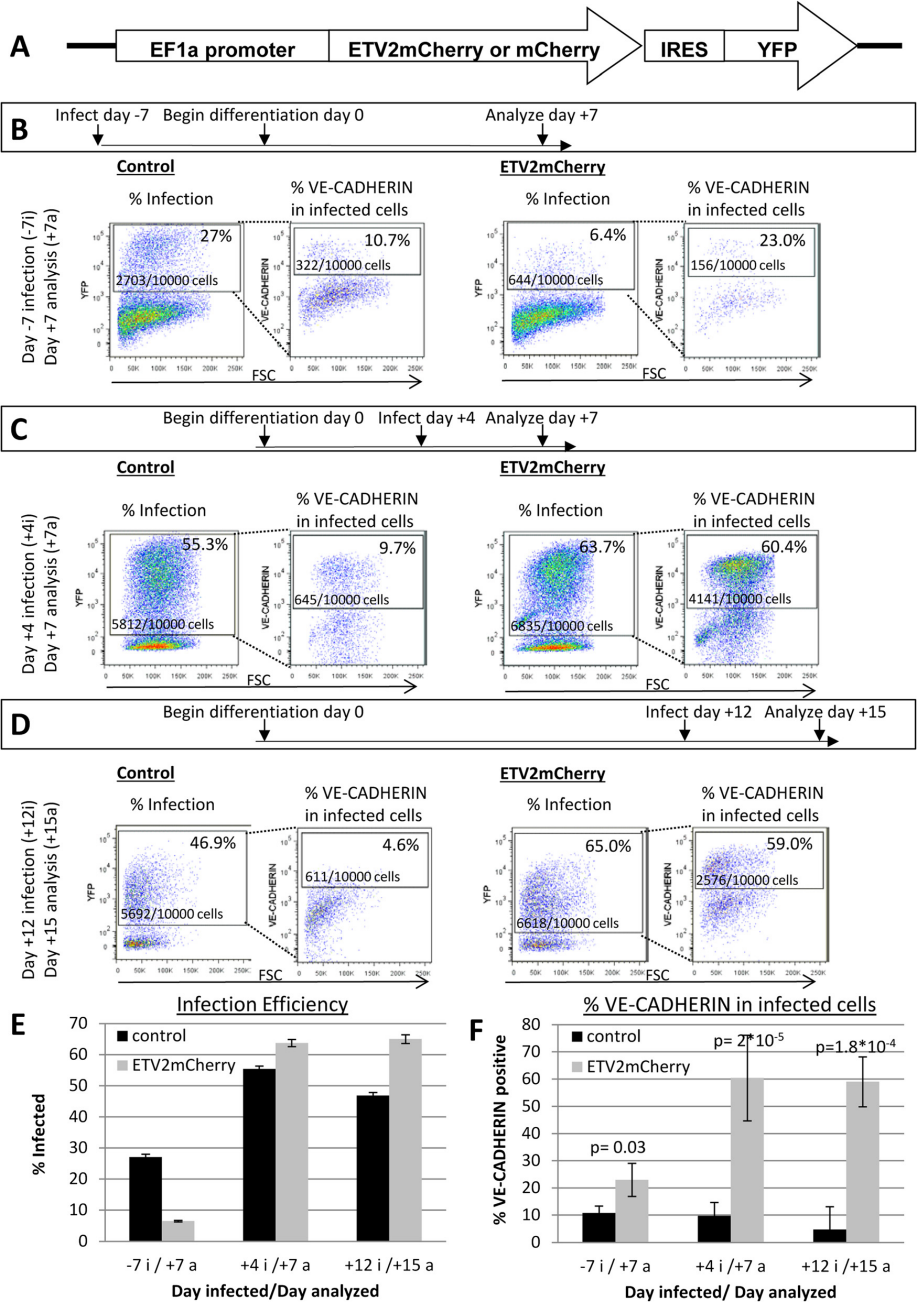
**Introduction of exogenous** ***ETV2*** **increases the percentage of endothelial cells generated during differentiation. (A)** Construct used to generate virus for introduction of *ETV2* to hESC. **(B–D)** Flow cytometry for YFP and VE-CADHERIN. Left panels of each subset show YFP expression in infected cells. Right panels of each subset show VE-CADHERIN expression within YFP+ population. Percentages represent an average from three **(B, D)** or six experiments **(C)** and cell counts represent and average from three **(B,D)** or four experiments **(C)**. Cells were infected/analyzed on days −7i/+7a **(B)**, +4i/+7a **(C)**, and +1i2/+15a **(D)**. Cell counts and percentages are calculated from cells gated to be non-debris, alive, and single cells. **(E)** Graphical summary of flow cytometry for infection efficiency in cells infected and analyzed on days indicated. Error bars indicate standard deviation. **(F)** Graphical summary of results of flow cytometry for VE-CADHERIN of YFP expressing virally infected cells. Error bars indicate standard deviation.

We constructed two lentiviral vectors to express either mCherry, as a control, or an ETV2-mCherry fusion protein (Figure 2A). Based upon transient transfection experiments, we found that the ETV2-mCherry fusion protein was localized to the nucleus but difficult to visualize by either microscopy or flow cytometry (data not shown). To ensure that we could identify virally infected cells, we co-expressed yellow fluorescent protein (YFP) with the mCherry or ETV2-mCherry proteins (Figure 2A). YFP expression was used as proxy for mCherry and ETV2-mCherry expression for the remainder of the experiments.

Previous studies in the murine system have examined the effect of exogenous *Etv2* on differentiating mESCs and demonstrated that up to 70% of the differentiating cells were responsive to exogenous *Etv2* [9]. mESCs were induced to transiently express exogenous *Etv2* concurrently with endogenous *Etv2* in differentiating embryoid bodies [9]. Experiments in hESCs have tested the ability of exogenous *ETV2* to induce endothelial cells from undifferentiated hESCs and from a FLK1+ subset of differentiated hESCs [12,17]. We wanted to examine whether the addition of *ETV2* at different stages of hESC differentiation would result in differing yields of endothelial-like cells. We infected cells either before differentiation (−7i/+7a) (Figure 2B) or at day 4 (+4i/+7a) (Figure 2C) of differentiation and analyzed the cells 7 days after the initiation of differentiation by flow cytometry for YFP and VE-CADHERIN expression. The infection efficiency of −7i/+7a cells was low in comparison to that of +4i/+7a, despite using a higher multiplicity of infection (MOI), 20 vs 2, respectively (Figure 2B,C,E). This agrees with previous publications demonstrating the low infection efficiency of hESCs [18,19]. In control virus-infected cells at each time point, a similar percentage of VE-CADHERIN positive cells (~10%) were observed compared to that in uninfected cells (Figure 2B,C,F). In the population infected with the *ETV2-mCherry* virus before differentiation, 22.9% of the cells were positive for VE-CADHERIN after differentiation, a twofold increase over the control (Figure 2B,C,F). This did not result in an absolute increase in endothelial-like cells as the slight increase in VE-CADHERIN-positive cells infected with *ETV2-mCherry* virus was counteracted by the decreased infection efficiency of these cells (Figure 2B). The apparent lack of response to *ETV2* in cells infected before differentiation may be due to the loss of cells that responded to *ETV2*. Cells that gained an endothelial-like phenotype in the 7 days after infection would have been excluded during the EB generation step of the differentiation protocol. *ETV2* administered during differentiation had a much more pronounced effect. Of the cells infected during differentiation, 60.4% were VE-CADHERIN positive on day 7 resulting in a sixfold increase over control cells (Figure 2B,C,F). This is comparable to what was seen in mESC culture [9]. The absolute number of cells that expressed VE-CADHERIN was significantly higher following *ETV2-mCherry* infection, increasing from an average of 645/10,000 cells in control conditions to 4,141/10,000 cells in *ETV2* overexpression conditions (Figure 2C).

Our previous work in zebrafish found that only a few cell types are responsive to exogenous *etv2* after 24 h of differentiation and unresponsive by 48 h [20]. Work performed *in vivo* in conditionally transgenic *Etv2* mice showed that cells committed to the somitic or neural lineages were unresponsive to exogenous *Etv2* [21]. Infection of adult human fibroblast and mesenchymal cells with an *ETV2* virus in combination with *FLI1* and *ERG1* viruses only modestly induced endothelial genes [17]. Based upon this knowledge, we anticipated that infection of differentiating hESCs with an *ETV2*-expressing virus at time points later than the peak of endogenous expression would result in decreasing percentages of cells responding to the exogenous signal. The cells were infected at day 12 of differentiation and analyzed for VE-CADHERIN expression 3 days after infection. Surprisingly, the surface expression of VE-CADHERIN was not significantly different in +12i/+15a *ETV2-mCherry-*infected cells from +4i/+7a *ETV2*-infected cells demonstrating that the cells had not lost responsiveness to *ETV2* as predicted (Figure 2D,F).

**Figure 3:**
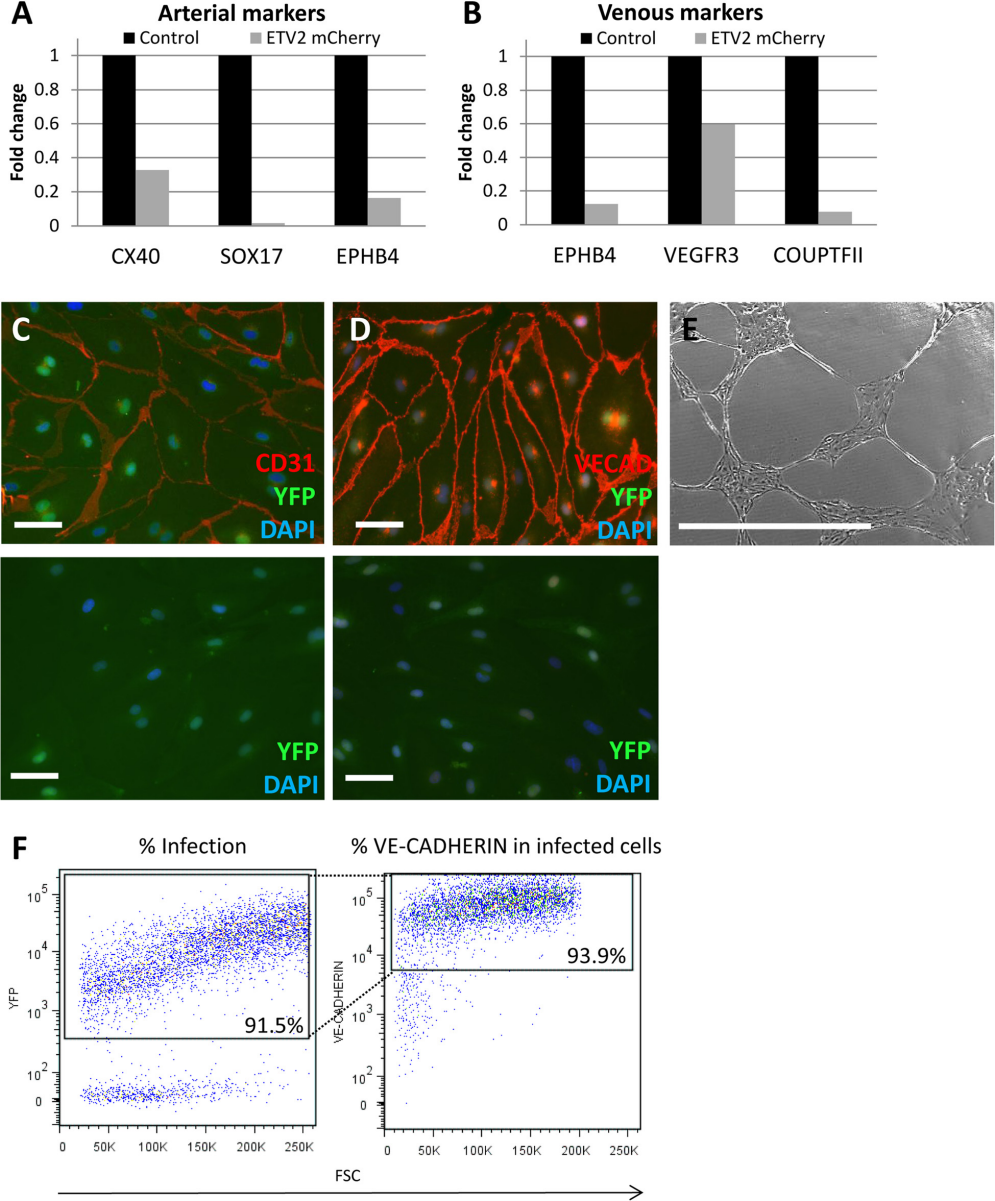
**Characterization of** ***ETV2*** **-induced endothelial cells. (A,B)** Semi-quantitative real-time PCR on sorted YFP+ VE-CADHERIN+ cells from day 7 of differentiation and infected in day 4 for arterial **(A)** and venous **(B)** markers. **(C,D)** Immunofluorescence against CD31 and VE-CADHERIN on sorted *ETV2-mCherry-*infected VE-CADHERIN-positive cells cultured for 7 days. Scale bar = 20 μm. Upper panels are anti-CD31 **(C)** or VE-CADHERIN **(D)** primary antibody and appropriate secondary. Lower panels, secondary antibody only. **(E)** Network formation on Matrigel of sorted *ETV2-mCherry* VE-CADHERIN positive cells grown for 7 days in culture. The cells were imaged 8 h after plating. Scale bar = 1 mm. **(F)** Flow cytometry on sorted *ETV2-mCherry*-infected VE-CADHERIN-positive cells cultured for 7 days. The cells were analyzed for YFP expression and VE-CADHERIN expression. Percentages are an average of two experiments.

We wanted to further characterize the endothelial-like cells that were generated from our viral transductions. Comparable numbers of *ETV2-mCherry* and control infected cells were sorted for YFP and VE-CADHERIN expression on day 7 of differentiation. RNA was isolated from these cells to determine the venous or arterial identity of these cells. Semi-quantitative real-time PCR performed on cDNA derived from this RNA demonstrated that cells infected with the *ETV2-mCherry* virus expressed lower levels of both arterial and venous markers (Figure 3A,B). This indicates that cells expressing very high levels of *ETV2* did not differentiate into arterial or venous endothelium and likely remain in an angioblast state. *In vivo* and *in vitro* data has shown that constitutive expression of *ETV2* causes endothelial cells to remain in a more primitive state [12,21].

Sorted YFP+ VE-CADHERIN+ cells were grown on gelatin in endothelial media supplemented with 20 ng/ml bFGF, 25 ng/ml VEGF165, and 10 μg/ml TGFβ inhibitor SB431542. The *ETV2*-infected cells proliferated faster than the control cells and maintained a uniform morphology (data not shown). An increased level of proliferation has been shown previously in cells constitutively expressing exogenous *ETV2* [17]. After 7 days of growth, the vast majority of *ETV2*-infected cells all maintained CD31 and VE-CADHERIN expression by immunostaining and flow cytometry (Figure 3C,D,F). These cells formed networks within 3 h of plating on Matrigel with optimal tube network formation at 8 h (Figure 3E).

Practical and ethical reasons prevent us from examining the timing and role of expression of *ETV2* in early human fetal samples. We have examined the timing of *ETV2* expression in differentiating human ESCs and found that its expression in relation to other developmental genes is similar to that found in the developing mouse *in vivo* and *in vitro*. In our method of infection during mesoderm-directed differentiation, 60% of the *ETV2* expressing cells were able to respond. This is similar to the level found in mESCs [9].

Clinical and preclinical trials are being performed that use endothelial cells from various sources to aid in the recovery from many vascular and cardiac conditions [22-24]. A common roadblock in these procedures is a lack of source allogeneic endothelial cells. Other clinical work is being performed to generate vascular structures that can then be seeded with endothelial cells prior to transplant into patients [25]. Use of induced pluripotent cells derived directly from the patient would allow for histocompatible graft material being available for transplant. Addition of *ETV2* is a viable strategy for increasing the yield of endothelial-like cells from these sources. Any cells transplanted into a patient would need to be derived through non-integrating viral or non-viral methods. These methods would result in a pulse of gene expression corresponding to the time of treatment. Using these methods would generate transient *ETV2* expression. This might allow the endothelial-like cells generated during *ETV2* expression, demonstrated in this report, to more fully differentiate into arterial and venous endothelial cells upon loss of *ETV2* expression. Recent work has shown that administration of *ETV2* and *GATA2* mRNA is able to convert hESCs to hematopoietic cells. Addition of *ETV*2 mRNA alone may be able to generate endothelial cells with an unaltered genome that could be used for transplantation into patients.

## Abbreviations

hESC: Human embryonic stem cells
mESC: Mouse embryonic stem cells
EB: Embryoid body
MOI: Multiplicity of infection
